# An Innovative Plate Concept for Rotational Guided Growth: A Porcine Pilot Study

**DOI:** 10.7759/cureus.58169

**Published:** 2024-04-13

**Authors:** Ahmed A Abood, Jan D Rölfing, Ahmed Halloum, Steffen Ringgaard, Jeppe S Byskov, Søren Kold, Ole Rahbek

**Affiliations:** 1 Department of Orthopaedic Surgery, Aarhus University Hospital, Aarhus, DNK; 2 Interdisciplinary Orthopaedics, Aalborg University Hospital, Aalborg, DNK; 3 Department of Interdisciplinary Orthopaedics, Aalborg University Hospital, Aalborg, DNK; 4 MR-Center, Aarhus University Hospital, Aarhus, DNK; 5 Department of Additive Manufacturing, Danish Technological Institute, Aarhus, DNK

**Keywords:** innovative implant, 3d print, guided growth, rotational deformity, maltorsion

## Abstract

Background

Rotational deformities in children are currently treated with an osteotomy, acute de-rotation, and surgical fixation. Meanwhile, guided growth is now the gold standard in pediatric coronal deformity correction. This study aimed to evaluate the feasibility of a novel implant intended for rotational guided growth (RotOs Plate) in a large porcine animal model.

Methodology

A submuscular plate was inserted on the medial and lateral aspect of the distal femoral physis of the left femur in 6 pigs. Each plate was anchored with a screw in the metaphysis and epiphysis respectively. The plates were expected to rotate the femur externally. The right femur acted as a control in a paired design. The animals were housed for 12 weeks after surgery. MRI scanning of both femora was performed before euthanasia after 12 weeks. Rotation was determined as the difference in the femoral version on MRI between the operated and non-operated femur after 12 weeks.

Results

External rotation in all operated femurs was observed. The mean difference in the femoral version on MRI between operated and non-operated femurs was 12.5° (range 9°-16°). No significant changes in axial growth were detected.

Conclusions

This study shows encouraging results regarding rotational guided growth, which may replace current invasive surgical treatment options for malrotation in children. However, further studies addressing potential secondary deformities are paramount and should be carried out.

## Introduction

Rotational deformities of long bones are currently corrected by osteotomies with internal or external fixation [1-6]. The concept of guided growth is state of the art for correction of valgus and varus deformities in the growing skeleton. Recently, the guided growth concept has also been advocated to correct rotational deformities because of the lesser invasive nature compared with corrective osteotomies [7-10]. A few studies have investigated oblique tension band plating with eight plates or similar implants in rabbits, i.e., small animal studies [11-14]. Recently, Metaizeau et al. published a study applying guided growth to correct torsion in humans using cable-connected cannulated screws, yielding promising results [15]. The concept of rotational guided growth in humans has furthermore been investigated by Paley and Shannon using oblique peripheral tethers [16]. All of these have proven to rotate long bones by guided growth. However, the nature of the plates applied may lead to growth retardation and changes in joint morphology, and they may not predictably rotate the bone [17,18]. However, these findings underline the possibility of applying guided growth to address torsional deformities in children.

An implant capable of rotating long bones in a controlled and predictable manner by guided growth is lacking. We designed a novel plate concept (RotOs Plate) for rotational guided growth, which was capable of rotating cadaverous femora in a controlled and predictable manner [19]. The plate is based on two curved oblique sliding holes interconnected at an angle of 105°. It is dependent on axial growth to obtain rotation and is thus unlikely to impair axial growth. This study aims to investigate the efficacy of the novel plate (RotOs Plate) in rotating porcine femora by guided growth.

## Materials and methods

Design and surgical procedure

**Figure 1:**
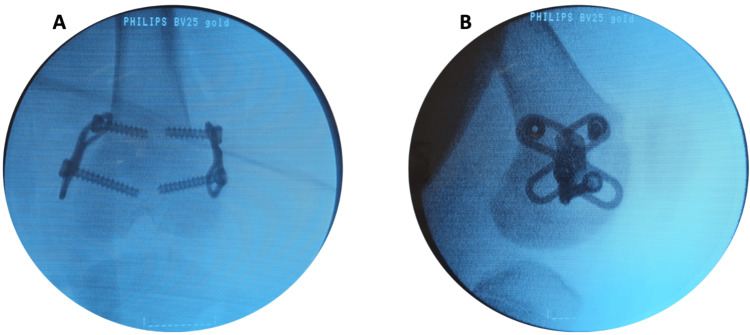
Intraoperative images. Intraoperative fluoroscopic images showing the insertion of the RotOs Plates in the porcine femur. Each plate is anchored with two 4.5 mm cannulated screws (DePuy Synthes): (A) coronal plane and (B) sagittal plane.

The study was carried out in a matched paired design. Six 12-week-old female pigs were included. The mean body weight was 43 kg (range 38-47 kg). The left femur was selected for surgical intervention. The right femur was left untreated in all animals. A submuscular plate (RotOs Plate) was inserted on the medial and lateral aspect of the distal femoral physis. The body of the plates was placed perpendicular to the physis in the sagittal plane with a sliding slot proximal and distal to the physis (Figure 1). Each plate was anchored proximal and distal to the physis using a 4.5 mm cannulated screw (titanium, DePuy Synthes). All surgeries were performed under general anesthesia with propofol (10 mg/kg/hour) and fentanyl (60 mg/kg/hour) as analgesics. All operated sites received a local anesthetic of 50 mg of Bupivacaine. The animals were housed and observed for 12 weeks after the operation, whereafter the plates were removed. MRI was subsequently performed to determine the left and right femoral versions. The difference in femoral version between the treated and nontreated femur was chosen as the primary outcome in this paired study.

RotOs Plate

**Figure 2:**
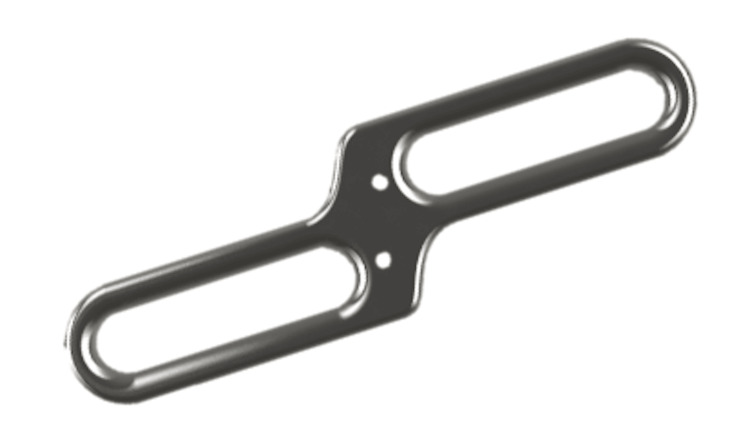
RotOs Plate. RotOs Plate concept plate with two sliding holes for screw fixation and two Kirschner-wire (K-wire) slots for temporary intraoperative fixation.

Two titanium (Ti6Al4V) plates per porcine were produced by additional manufactured, i.e., 3D printed by Danish Technological Institute, Aarhus, Denmark. RotOs Plate consists of two oblique and curved sliding slots interconnected at an angle of 105° through a body with two slots for temporary Kirschner-wire fixation (Figure 2). A screw was inserted proximally in each sliding slot. The length of the sliding holes was 13 mm. The distance between the two screws in the transverse plane, with the body aligned to the femoral axis, was 17 mm, as designed by the plate. Similar plates were applied in cadavers achieving rotation during axial growth [19].

MRI and measurement of the femoral version 

**Figure 3:**
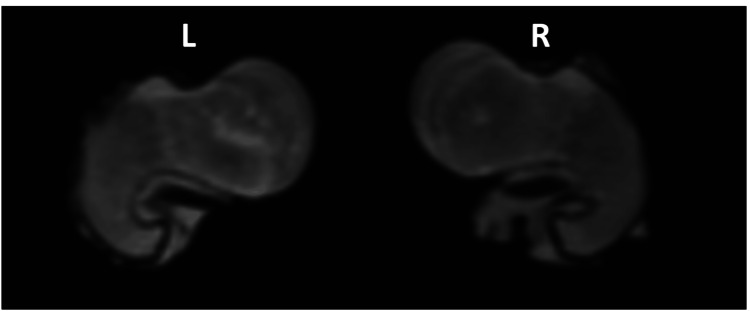
MRI image of the femoral neck in the operated and non-operated femur with both femoral condyles aligned parallel to the scanning table showing the difference in femoral anteversion: L, left operated femur; R, right non-operated femur.

MRI with a 3.0 Tesla scanner (Siemens Skyra) was performed 12 weeks after surgery. T1 and T2 sequences were obtained. The femoral version was determined as the angle between an axis through the femoral condyles and the axis of the femoral neck in the axial plane. The difference in the femoral version between the right and left femur was assigned as obtained rotation (Figure 3).

**Figure 4:**
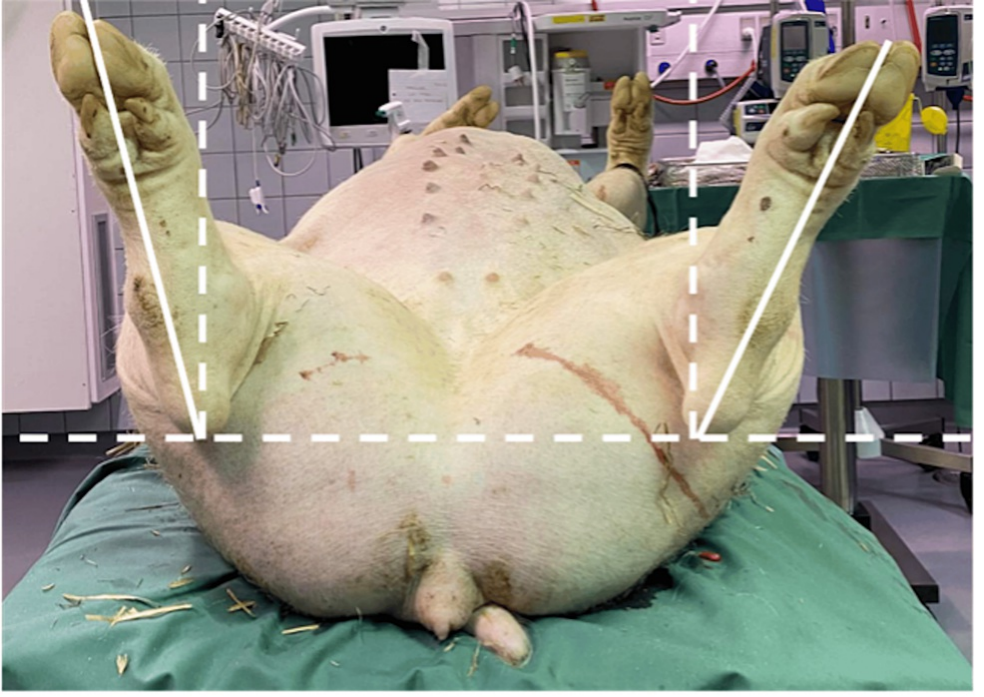
Differences in clinical rotation of the hind limbs are shown in a clinical image obtained on the leveled table.

Clinical evaluation of the difference in torsion was done by measuring the angle between the foot and a perpendicular line to a line connecting the calcaneal tubercules. Measurements were done with all animals lying straight and supine on a leveled table (Figure 4).

The femoral length was measured in the coronal plane from the top of the femoral head to the bottom of the medial femoral condyle.

Statistics

All calculations were conducted using Stata 16 software (Stata Statistical Software: Release 16, StataCorp LLC, College Station, TX). Paired t-tests were applied to determine the *P*-values. *P*-values ≤ 0.05 were considered statistically significant. Data are presented as mean (95% confidence interval) or median (range).

## Results

**Figure 5:**
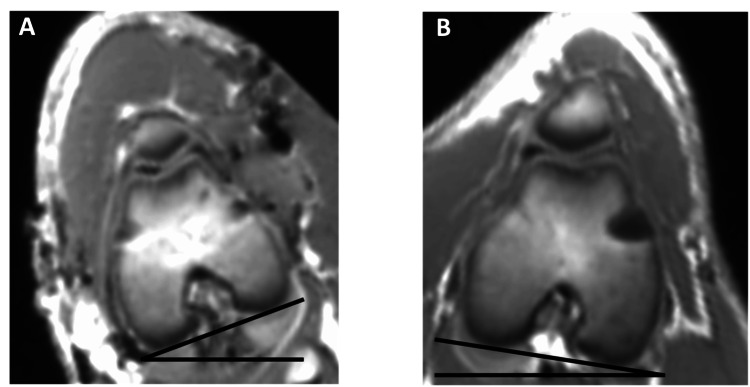
Rotational difference of the distal femur on MRI: (A) left operated femur and (B) right non-operated femur. MRI images of the distal femora in the operated and non-operated femur showing the difference in the femoral version. For illustrational purposes, both femoral necks are aligned parallel to the axis of the table revealing the difference in the distal femur.

All animals tolerated the surgeries well and were ambulatory on the first postoperative day. One postoperative wound deficiency was observed, which required re-suturing. No infections or other complications were identified, and no animals were sacrificed before the end of the study. In two operated femora, only one of the two inserted plates was anchored proximal and distal to the physis as intended, resulting in only one plate guiding the growth. All operated left femurs were externally rotated and compared with the contralateral control on MRI (Figure 5). Clinical examination confirmed the rotation (Figure 4). The mean difference in the femoral version was 12.5° (range 9°-16°). Clinical examination indicated a torsional difference of 10.3° (range 7°-14°). The operated femur was 2.7 mm (-0.4 to 5.7) shorter than the non-operated femur (Table 1).

**Table 1:** Measured rotational differences. Results showing measurements of torsional and length differences by MRI and clinical evaluation. The two animals marked with * only had one RotOs Plate spanning the physis.

	Difference in the femoral version MRI (°)	Difference in the femoral version clinical examination (°)	Difference in length MRI (mm)
Animal 1	12	9	- 1
Animal 2*	9	7	0
Animal 3	15	14	3
Animal 4*	10	12	4
Animal 5	16	9	3
Animal 6	13	11	7

## Discussion

This is the first study investigating the in vivo efficacy of the RotOs Plate to perform rotational guided growth in a large animal model. The efficacy of the plate to create the intended external rotation was confirmed in all six animals. This encourages the possibility of applying such a plate concept for rotational guided growth in case of a torsional malalignment in children. Although the analyses performed suggest an obtained rotation of 12.5°, the actual obtained rotation may differ as we lack baseline imaging. Hence, we assumed a symmetrical femoral growth and version in all animals, which may not be the case. We recently published a cadaverous study investigating the mechanical properties of the plate concept, which demonstrated a possible rotation of approximately 20° [19]. The difference in obtained rotation may be explained by several factors. Most importantly, measurement of the porcine femoral version is difficult due to the short femoral neck. Hence, these results should be understood as binary results, illustrating the efficacy of the plates in performing rotational guided growth and not quantifying the actual rotation caused by the plate insertion. Moreover, the possibility that soft tissue and/or the periosteum might play a role, reducing the actual rotational ability of the plates should not be ignored. Another likely explanation could be insufficient axial growth to fulfill the rotational potential of the plates. A previous study performed in similar animals indicates an axial growth of approximately 2 cm in the whole femur in a similar observation period [20]. This is comparable to the axial distraction in the cadaverous study, why axial growth of this magnitude was expected to be sufficient [19]. However, the plates were only spanning the distal femoral physis, meaning that growth in the proximal physis did not affect the plates. The difference in version would likely have been larger if observation time had been increased as rotation occurs during axial growth in the distal femoral physis. However, the sole purpose of this study was to determine whether the plates were able to create a torsional difference, rather than the size of it. 

Despite these encouraging results, it is important to further investigate the possible creation of a secondary deformity in all planes due to the guided growth. Especially taking into consideration, that one functioning plate in two animals was sufficient to create a torsional difference. This raises the question if one plate with long screws might be adequate to perform rotational guided growth or if this will additionally create a secondary deformity or a change in joint morphology. Unfortunately, it was not possible to determine whether a significant secondary sagittal or coronal deformity was created in all animals due to the lack of baseline scans. We did not observe a significant difference in femoral length comparing the operated to the non-operated side. However, this is something to take into serious consideration in prolonged plate insertion, as the plates are likely to resemble eight plates, intended for leg length discrepancy (LLD) when the rotational potential of the plates has been achieved. In this case one should be aware of both decreased axial growth and central overgrowth [17]. Another critical issue to be aware of is the possibility of the rebound phenomenon [21-23]. This was not investigated in this study as all animals were euthanized after the removal of the plates. This phenomenon is more likely to be associated with the concept of rotational guided growth rather than the choice of implant or technique. Other studies have investigated the concept of rotational-guided growth. The study by Metaizeau et al. is arguably the most noticeable as it was performed in humans using an interesting technique [15]. The presented results are truly impressive regarding rotational guided growth, but the applied technique represents a tension-band-like implant immediately upon insertion. A concern however remains that this may decrease axial growth causing LLD. We have intended to deal with this issue by introducing the sliding slots in the plate, which allows the plate to travel in the axial plane during rotational guided growth and thus not impair axial growth. 

The main limitations of this study are the lack of a baseline scan and the low number of animals. This limited our analyses to solely assessing the torsional difference between the operated and non-operated femur, excluding proper analyses regarding the size of the torsional difference, rebound phenomenon, secondary deformities, and growth inhibition. The study was carried out during the COVID-19 pandemic, which unfortunately limited access to the experimental facilities at our institute. We have already initiated other and more comprehensive studies to consider our known limitations including an optimized design of the plate to avoid having animals with only one plate spanning the physis.

## Conclusions

The efficacy of RotOs Plate to achieve rotation in the intended direction has been proven. The need for further investigation looking into the accuracy of the predicted to the achieved rotation, as well as its efficacy to maintain the longitudinal growth, rebound phenomenon, and especially secondary deformities, is paramount.

The use of rotational guided growth is likely to increase in the future being a more tolerant technique when compared to the current alternatives, which likely may increase the spectrum for indications.
